# Advancement flap technique for a rare complex anal fistula with synovial cyst at the ischial tuberosity

**DOI:** 10.1007/s10151-022-02578-0

**Published:** 2022-03-02

**Authors:** C.-F. Xiao, Y.-Q. Ding, Y.-B. Pan, Y.-Q. Cao, C. Wang, Y.-B. Yao

**Affiliations:** Department of Anorectal Surgery, Longhua Hospital, Shanghai University of Traditional Chinese Medicine, Shanghai, China

**Fig. 1:**
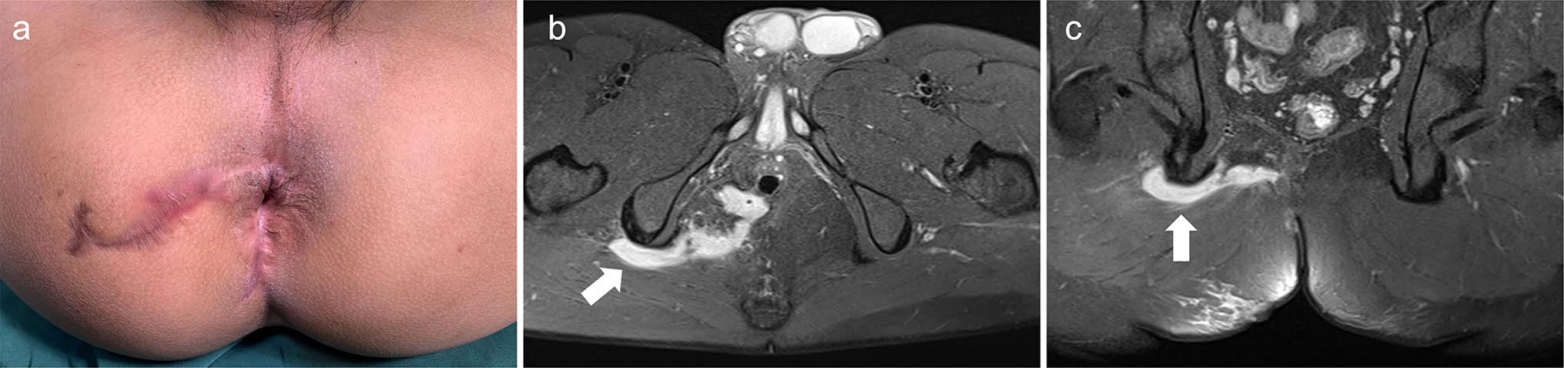
A 28-year-old man with a history of three operations for complex anal fistula presented to our department complaining of perianal discomfort. **a** Physical examination revealed 2 old surgical scars around the anus. **b**–**c** Perianal magnetic resonance imaging (MRI) was used for preoperative diagnosis and showed that the fistula passed through the anal sphincter to the outer lower edge of the right ischial tuberosity (white arrow)

**Fig. 2:**
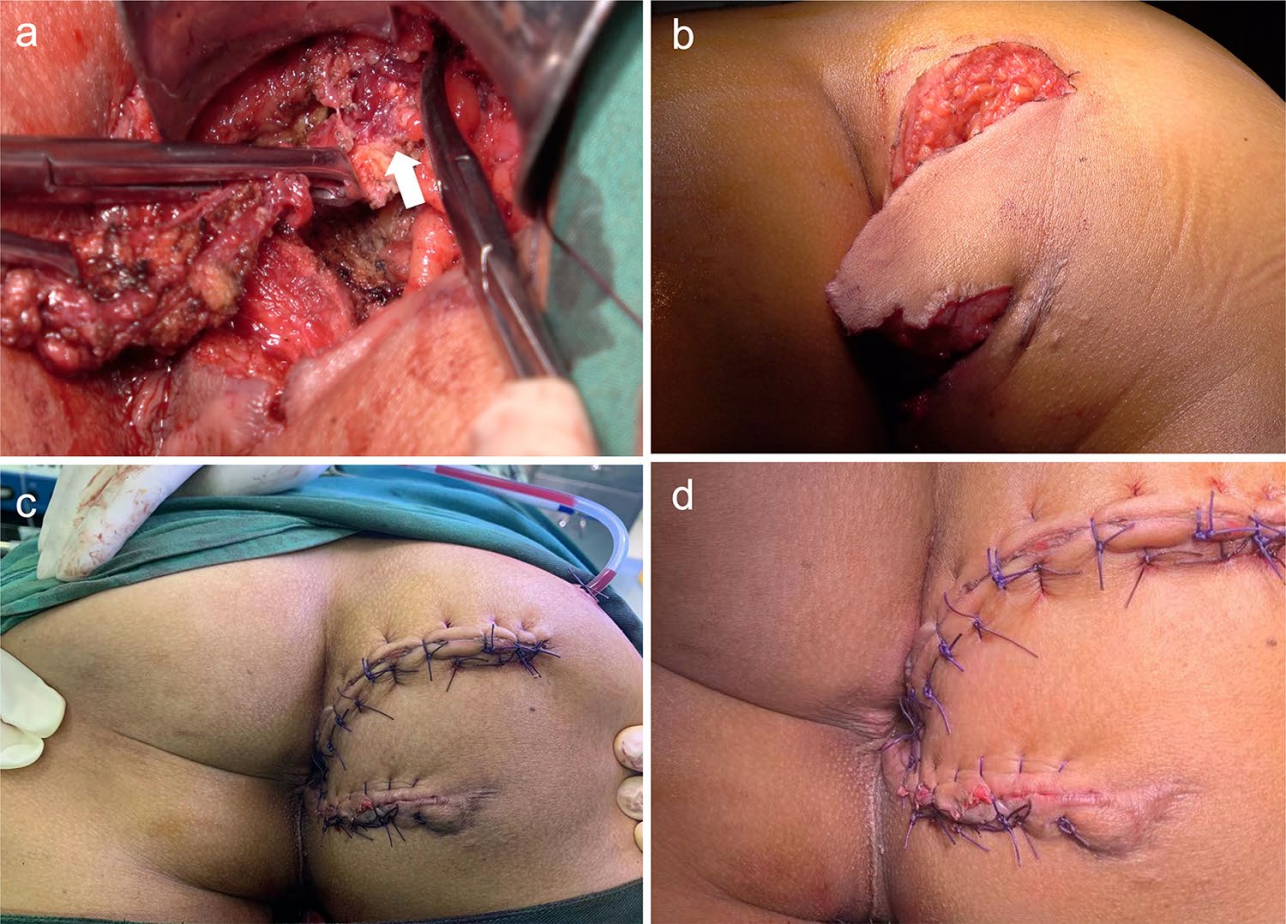
An internal opening was found above the dentate line at 7 o’clock in the lithotomy position. **a** Two surgical scars were cut during the operation, and cystic wall like tissue (white arrow) was found at the ischial tuberosity, which was consistent with the MRI findings. Based on MRI and intraoperative findings synovial cysts of the ischial tuberosity were suspected, and postoperative pathology findings supported this diagnosis. **b**–**d** The advancement flap was freed to cover the internal opening without tension, after the lesion was completely removed and the gluteus maximus was reconstructed

**Fig. 3:**
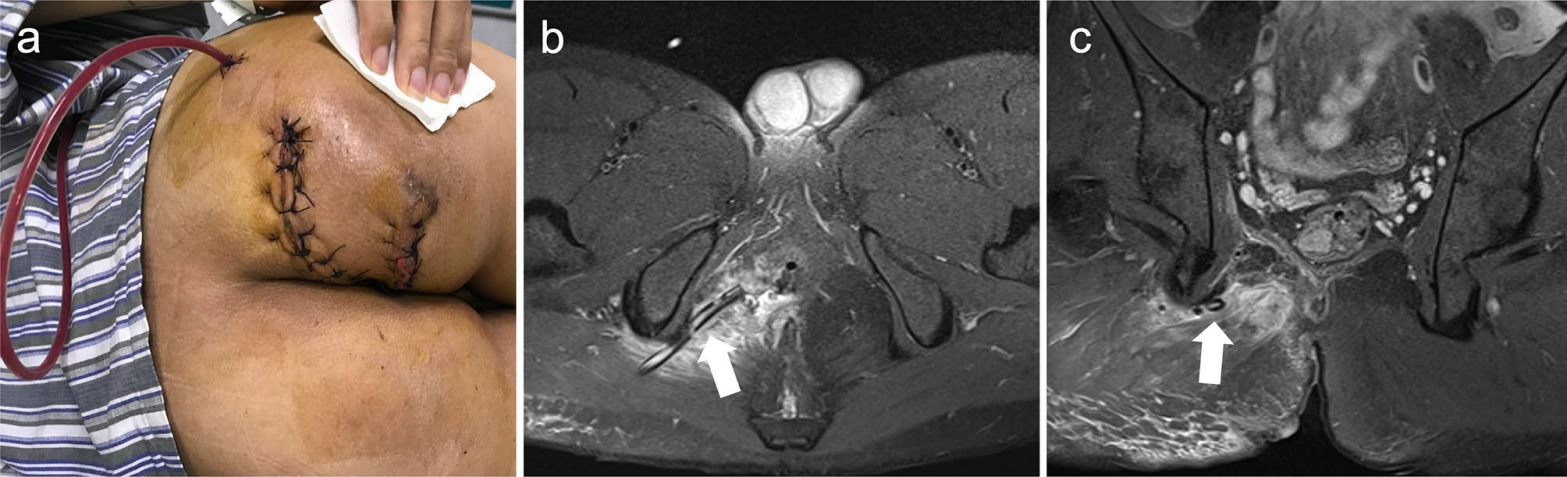
After reexamination of MRI on postoperative day 8, it was found that there was no obvious effusion, and then the drainage tube was removed. **b**–**c** MRI on postoperative day 8 (white arrow: drainage tube)

**Fig. 4:**
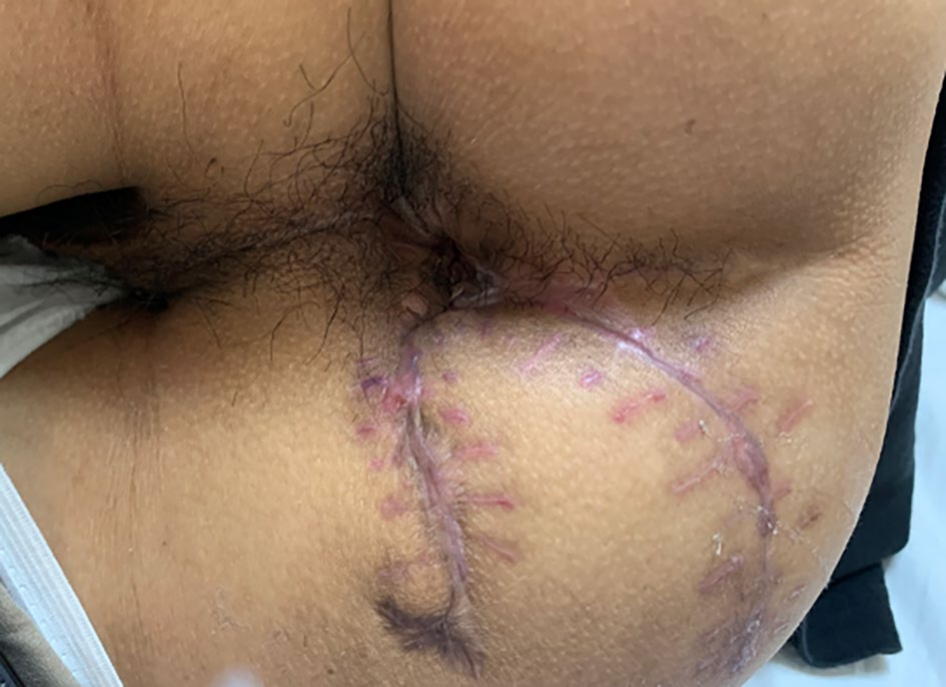
Wound healing 2 months after the operation

**Fig. 5:**
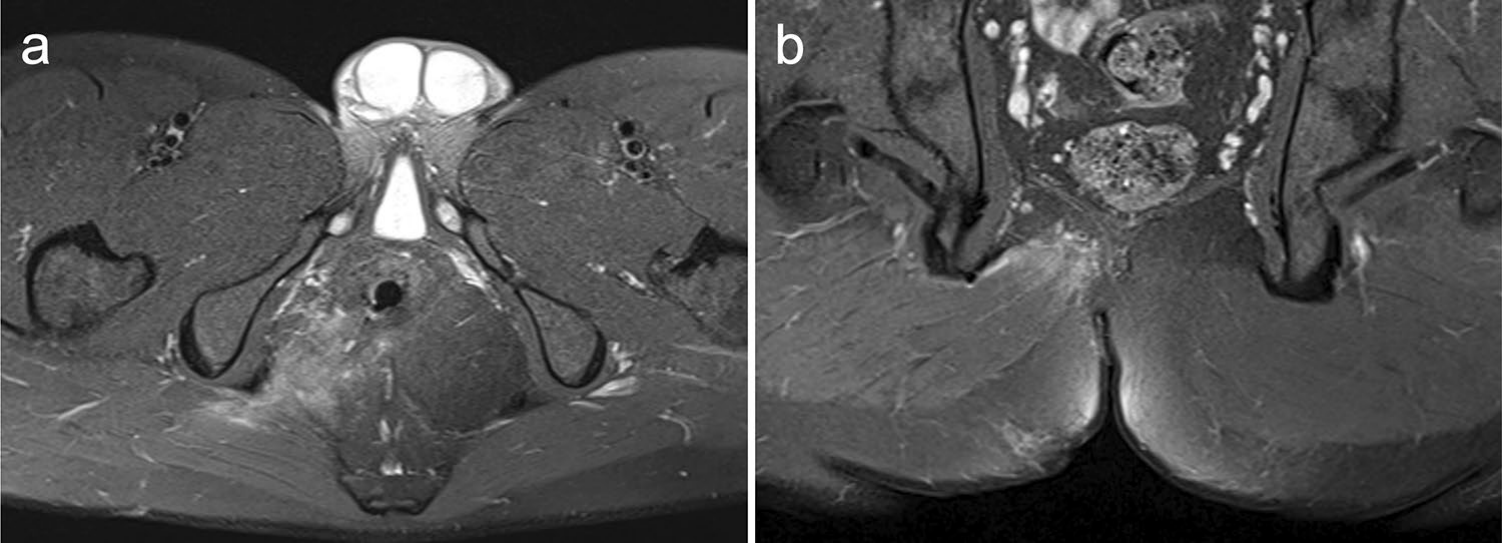
MRI 5 months after the operation. Imaging showed excellent healing

Complex anal fistula with synovial cyst at the ischial tuberosity is a very rare condition. The key point in the operation is to protect the anal sphincter and completely peel off the cyst. Here, we report a successful case of closing the internal opening with the advancement flap technique and completely stripping the cyst (Figs. 1, 2, 3, 4, 5).

